# Supplementary material for: Are carbon emissions trading and green financial instruments synergistic? -Comprehensive quantitative research based on content analysis
Source: PLoS One. 2024 Mar 7;19(3):e0298601. doi: 10.1371/journal.pone.0298601 (PMC10919601; doi:10.1371/journal.pone.0298601)
Supplement: S1 Data — (ZIP) [file pone.0298601.s001.zip › Data/地级市Z_F工作报告.zip!/┴╔─■/╙¬┐┌╩╨/└┤╫╘╣1⁄2╓┌║┼í╛┬φ┐╦╩2╛▌═°í┐_2010.docx]

各位代表：

　　现在，我代表市政府向大会报告工作，请予审议，并请市政协委员和列席会议的同志提出意见和建议。

　　一、2009年全市人民攻坚克难，圆满完成了经济社会发展的任务目标

　　刚刚过去的2009年，是极不平凡的一年。一年来，在市委的正确领导下，在市人大、市政协的监督支持下，市政府紧紧依靠全市人民，积极应对金融危机带来的多重考验，克服前所未有的困难，圆满完成了市十四届人大二次会议确定的各项任务。地区生产总值预计实现822亿元，增长20%；财政一般预算收入57.1亿元，增长42.8%；全社会固定资产投资800亿元，增长47.3%；规模以上工业增加值522.1亿元，增长25.6%；社会消费品零售总额211.4亿元，增长18.5%；城市人均可支配收入15940元，增长11%；农民人均纯收入7798元，增长12.3%；全市金融机构各项本外币存贷款余额分别为835.8亿元和681.1亿元，增长31.8%和43.4%；全社会用电量和工业用电量首次突破100亿千瓦时。各项社会事业取得了新的进展。

　　（一）调整优化结构，推动产业转型

　　以培育现代农业为重点，加快农村经济发展。农业总产值实现116.5亿元，增长10.6%。大力发展高效农业，创建国家、省水稻高产示范区，最高单产和平均产量均列全省第1位，粮食总产量达到73.3万吨。加快推进农业产业化，新上1000万元以上农业产业化项目31项，新增“三品”认定面积11万亩。“营口大米”获中国驰名商标，“盖州苹果”成功注册国家地理标志证明商标。积极开拓农产品市场，新增农民专业合作社271家，新增订单农业面积10万亩。成功举办了营口市首届农业博览会暨农商对接会，签订协议合同额1.25亿元。农村基础设施和生活环境得到改善，新建农村饮水安全工程71处，生物质气化集中供气工程10项，改造农村无害化卫生厕所2500座，完成农村沼气池3000户。

　　以发展新兴产业为目标，提升工业经济整体水平。工业经济保持快速增长。全市规模以上工业总产值实现1676.9亿元，增长23.8%，新增规模以上工业企业207户。六大主导产业实现工业增加值416.2亿元，增长24.7%，占全市工业总量的79.7%。科技创新能力不断增强。在巩固镁质材料等传统产业的同时，积极发展先进装备制造、新能源、新材料、电子信息产业，中冶京诚一、二期、晶晶光电等项目投产，新东北电气、东方光大等项目开工建设。实施技术创新项目141项，高新技术产业增加值实现92.5亿元，增长51.4%。成功并购加拿大麦格诺拉公司等国外科技型企业，引进海外科技研发团队16个。科技研发平台进一步拓展。大连理工大学营口研究院落户沿海产业基地，中国矿业大学科技园孵化基地正式签约，高新区渤海科技城建设顺利推进，市级以上企业技术中心达到95个。

　　以重大项目建设为牵动，加快服务业发展步伐。服务业增加值完成275亿元，增长16.5%。市场体系日趋完善，乐购超市、相和购物中心等一批重点项目开工建设，服务业项目投资达322亿元，增长95.6%。房地产业创历史最好水平，商品房销售额实现101.6亿元，增长101.3%。旅游业发展实现新突破，天沐温泉城等一批牵动力强的重点项目投入使用，双台子温泉城和星级酒店项目全面开工，成功举办首届中国（营口）国际海滨温泉节。全年旅游总收入实现103亿元，增长67%。我市被第二届中国国际休闲发展论坛评为“2009中国十大特色休闲城市”。金融环境进一步优化，融资担保平台建设扎实推进，支撑发展能力显著提高。当年域内银行新增贷款206亿元，信贷投放创历史最高水平。营口银行沈阳分行和盛京银行、大连银行营口分行正式开业，全市域内外银行贷款规模突破900亿元。

　　（二）加快沿海开发，推进规划建设

　　围绕打造百里滨海生态城、百里沿海产业带、百里临海景观带，积极推进沿海经济带开发建设。坚持科学论证、高标准制定城市规划。聘请国内外一流的设计单位和专家，制定营口沿海带形城市总体规划。同时，对北海新区、营东新城、仙人岛旅游度假区、河海新西市进行规划，为高起点、高标准建设百里城市带奠定了基础。

　　港口功能不断提升。仙人岛港区总体规划获国家批复，30万吨级原油码头投产使用。鲅鱼圈港区A港池5＃、6#泊位建设全部竣工，保税物流中心正式封关运行。营口港与神华、中海、吉煤等六大集团的战略合作全面展开，服务功能进一步延伸。成功接卸东北口岸第一艘8530标箱、30万吨级原油、30万吨级矿石船舶。港口吞吐量完成1.76亿吨，增长16.7%；集装箱运量253.7万标箱，增长24.6%。

　　园区开发建设步伐加快。沿海经济带累计开发土地128.6平方公里，完成投资673.6亿元，签约项目418项。开发区龙头作用日趋显现，新开工亿元以上项目58项，各项主要经济指标继续保持30%以上的强势增长。沿海产业基地、仙人岛能源化工区、高新区、中小企业园基础设施进一步完善，项目承载能力显著提升。

　　（三）狠抓项目建设，提高开放水平

　　扎实开展“项目建设年”活动，积极培育新的经济增长点。实施“5227”工程，全年开工建设1000万元以上项目799项，其中亿元以上项目269项。项目建设投资总额715亿元，增长50%。华能营口热电、五矿宽厚板等一批重点项目竣工投产，可口可乐等一批项目开工建设。

　　转变招商方式，外引内联成效显著。采取主题概念招商、产业集群招商等方式，推出了“微电光电产业园”、“营口北海新经济区”等一批招商主题，招商引资实现新突破。全年批准外资项目69项，合同外资额11.2亿美元，增长22.3%。其中，投资1000万美元以上项目42项。实际利用外资完成5亿美元，增长56%。引进内资项目410项，到位资金380亿元，增长72.7%。

　　采取积极措施，遏制外贸出口下滑势头。制定了出口产品财政奖励政策，建立重点出口企业联动机制，培育新的外贸出口产业，克服镁制品、钢铁、纺织服装等重点出口产品国际市场萧条带来的不利影响，外贸出口急剧下滑势头一定程度上得到遏制。全年实现外贸出口12亿美元，下降28.8%。

　　加大政策扶持和引导力度，保持经济平稳健康运行。把保增长的重点放在保企业上，及时制定了促进企业稳定运行的一系列政策措施，重点解决生产中遇到的水、电、气和资金等突出问题，对困难企业降低和缓征社会保险金、价调基金和河道维护费。列入中央和省政策性产业项目183项，争取国债资金和省专项补助资金4.26亿元，地方债券3.3亿元。

　　（四）着力改善民生，促进社会和谐

　　强化就业和再就业工作，进一步完善社会保障体系。城镇实名制就业8.8万人，普惠制就业培训7.2万人，转移农村剩余劳动力2.8万人，“零就业家庭”动态为零。企业养老保险参保人数达到43.2万人，企业离退休人员养老金实现按时足额发放。城镇职工和居民医疗保险覆盖面进一步扩大，新农合参合率达到99%。全年开工建设回迁楼211万平方米、廉租房5.33万平方米，改造农村特困户危房465户。投入扶贫开发资金1992万元，1.5万农村贫困人口实现稳定脱贫。城乡低保实现动态管理下的应保尽保。

　　加强城乡基础设施建设，改善优化人居环境。建设渤海大街东段等公路项目66项，其中农村公路项目19项。东部污水处理厂、医疗垃圾处理厂等基础设施项目投入使用，营口机场等重大项目建设有序推进。民兴河改造等“五大惠民工程、十件实事”当年任务全面完成，跨年度工程完成序时进度。加大重点地区环境整治力度，治理大石桥境内硼泥堆放场5处，大清河流域水质得到改善。城市绿化覆盖率提高2.3个百分点，我市被省政府命名为省级园林城市。

　　推动经济社会协调发展，社会事业全面进步。营口大学园、省实验学校如期开学，开创了我市教育史上新的里程碑。努力改善办学条件，投入1.2亿元对城乡47所中小学操场实施改造。推进文化体制改革综合试点工作，完成专业剧团和新华书店转企改制。成功举办了“2009年中国营口望儿山母亲节”系列活动，并荣获“全国节日百强庆祝活动”称号。有效开展甲型H1N1流感等传染病防治，疾病预防控制和医疗救治水平明显提高。群众体育和竞技体育蓬勃发展，在第十一届全运会上，我市金牌数量和团体总分均列全省第4位。人口和计划生育服务能力进一步增强，人口自然增长率控制在1.35％。以内。加强重点民生工程建设，新建成的市光荣院、救助站和养老院均达到国内一流水平。广播电视、档案、民族宗教、老龄、慈善、妇女、儿童工作、残疾人事业和双拥共建、民兵预备役等各项事业取得了新成绩。

　　加强社会治安综合治理，保持社会和谐稳定。进一步加强城市巡防体系建设，严厉打击各种违法犯罪活动，命案侦破率100%。认真做好国庆安保工作，积极推进平安建设。深入开展“解难题促和谐”和“积案化解年”活动，全市信访量下降10%。高度重视并切实强化安全生产工作，建立了季调度制度，不断加大安全隐患整治力度，全年没有发生重特大安全生产事故。

　　（五）加强自身建设，改善政务环境

　　加强民主法制建设，促进科学民主决策。认真贯彻落实市人大及其常委会的各项决议决定，自觉接受人大法律监督、工作监督和政协民主监督，积极听取各民主党派、工商联、无党派人士和各人民团体的意见。全年办理人大代表建议204件、政协委员提案194件。畅通群众诉求渠道，人民群众的合理诉求得到有效解决，全年群众投诉办结率86%。坚持依法行政，不断规范行政执法，行政行为逐步步入法制化、规范化轨道。

　　转变工作作风，打造良好服务环境。新一轮政府机构改革顺利实施。扎实推进政务公开和效能建设，有效保障了人民群众的知情权、诉求权和监督权。加强廉政建设，进一步强化对工程项目招投标、国有土地使用权招拍挂和政府采购的监管，加强对重点专项资金和重大项目建设资金的跟踪审计，纠正损害群众利益的不正之风，政府公共管理和服务水平不断提高。

　　各位代表，过去的一年，营口经济社会发展的成果来之不易，这是市委正确领导的结果，是市人大、市政协和社会各界监督支持的结果，是全市人民同心同德、共克时艰、努力拼搏的结果。在此，我代表市政府向各级人大代表、政协委员、各民主党派、工商联、无党派人士、各人民团体，向离退休老领导、老同志，向驻营解放军、武警部队官兵和中省直单位，向辛勤工作在各条战线的同志们，向所有关心和支持营口建设的朋友们，表示衷心的感谢并致以崇高的敬意！

　　在肯定成绩的同时，我们清醒认识到，在经济社会发展的进程中，还存在一些突出问题，主要是经济总量偏小，产业集群培育滞后，新兴产业发展较慢，服务业规模不大，区域经济发展不平衡；面临的就业再就业、社会稳定、社会事业发展、财政收支、环境和生态保护等工作压力较大；政府机关仍然存在工作思路不宽，办事效率不高，服务意识不强等问题，需要我们采取有力措施认真加以解决。

　　二、2010年以产业升级和城市转型为重点，努力实现创新和发展的新突破

　　今年是实施“十一五”规划的最后一年，做好今年工作对全面完成“十一五”规划，实现“十二五”发展的高起点开局，加速推进沿海经济强市建设至关重要。当前，宏观经济环境趋向好转，特别是辽宁沿海经济带上升为国家战略，营口迎来了更加广阔的发展空间。立足新起点，抢抓新机遇，实现新跨越，是全市人民的共同愿望，也是政府必须承担的历史责任。

　　全年经济社会发展的总体思路是：坚持以邓小平理论和“三个代表”重要思想为指导，全面贯彻党的十七大、十七届三中、四中全会、中央经济工作会议和省委十届九次全会精神，深入落实科学发展观，按照市委十届十一次全会提出的“保增长、调结构、促开放、惠民生”的总体要求，围绕产业升级和城市转型，坚持以港兴市、工业强市、科技立市战略，加快工业新型化、服务业现代化、农业产业化和沿海区域农村城市化步伐，着力改善民生，促进社会和谐，实现经济社会又好又快发展。

　　全市经济社会发展的主要目标是：地区生产总值完成1000亿元，增长20%；全社会固定资产投资1080亿元，增长35%；规模以上工业增加值652亿元，增长25%；社会消费品零售总额253亿元，增长20%；财政一般预算收入71.5亿元，增长25%；城市人均可支配收入和农民人均纯收入分别达到17850元、8968元，增长12%、15%；城镇登记失业率控制在4%以下；人口自然增长率控制在1.78％。以内。

　　为实现上述目标，重点抓好以下七个方面工作：

　　（一）保持经济持续增长，确保实现“双千亿”目标

　　强化投资对经济增长的拉动作用。继续开展“项目建设年”活动，实施“7321”工程，即全年开工建设1000万元以上项目700项，其中亿元以上项目300项，新增规模以上工业企业200户，固定资产投资1080亿元。坚持以项目建设为核心，以外引内联为重点，突出主题概念招商和产业集群招商，加强政策引导和项目包装，提高项目建设的针对性和实效性。全年实施“四个一批”项目100项，实际利用外资6.5亿美元，增长30%；引进内资500亿元，增长31.6%。积极推进新加坡食品工业园、金宏矿业金属镁、中冶京诚三期工程建设，规划一批龙头项目和产业集群项目，确保项目建设实现新突破。

　　解决扩大内需和外贸出口两个突出问题。继续实施积极的消费政策，改善消费预期，优化消费结构。认真落实家电下乡补贴政策，促进农村消费水平进一步提高。鼓励发展消费信贷，积极培育消费热点，强化消费拉动。打好外贸出口攻坚战。用好用足国家鼓励出口相关政策，优化出口产品结构，扩大镁制品和纺织服装出口，增加轻工、化工和机电产品出口，拓展农副产品出口。实施名牌战略，重点推进一批名牌出口产品，提高国际市场竞争力。全年外贸出口确保完成14.4亿美元，增长20%。

　　改善优化金融环境。建立健全政府、银行、企业合作机制，引导金融机构增加信贷投放，确保存贷比高于全省平均水平。进一步加强融资平台、担保体系建设，拓宽融资渠道，实现资金筹措和融资方式的新突破。积极引进金融机构，年内力争招商、广发、中信、浦发四家银行在营设立分支机构。抓紧推进企业上市工作，积极引入创业投资和风险投资，加快组建小额贷款公司和村镇银行，全年新增15家小额贷款公司，进一步解决中小企业融资难问题。加强税收管理，增强全民纳税意识，打造和谐的税收环境。

　　（二）以实施沿海“三个百里”规划为重点，加速推进沿海经济带开发建设

　　建设百里滨海生态城。按照“东扩、西改、南进、北转”的发展思路，加快老城区改造建设步伐，始终不渝推动老城区繁荣发展。老边区重点推进营东新城建设，加快城中村改造，逐步实现城乡一体化。西市区要高起点做好城区规划，高标准推进平房区改造和河北区片开发，建设“河海生态宜居区”和“现代商贸休闲区”，重现西部繁荣。站前区以旧小区改造和北部城区功能转型为重点，加快发展流通服务业，完善综合配套，塑造老城中心区新形象。根据“滨海、带形、生态、组团”的城市规划，加大基础设施建设力度，坚定不移推进滨海生态城建设。按照“工业新城、老城新区”的定位，沿海产业基地要加速商贸中心区建设，着力打造具有国际影响力的新城区。以“沿海带形城市中心区”的定位，加快北海新城的基础设施和行政公共设施建设，确保全年投资100亿元以上。按照中等城市规模开发建设，打造开发区“渤海明珠”城市品牌，高标准推进南部新城区和望海新区建设，进一步做大城市规模，建设辽宁沿海经济带科学发展示范区。进一步完善规划，充分利用仙人岛能源化工区良好的自然资源，加快白沙湾黄金海岸开发，高标准规划建设滨海旅游休闲度假区。

　　打造百里沿海产业带。按照区域协调、产业升级和科学发展的原则，坚持以项目建设为核心，加快沿海“三园、二区、一基地”建设，使其成为营口发展的战略平台。港口要加快粮食码头、25万吨航道工程和疏港公路、铁路等基础设施建设，加大国际航线开发力度，进一步提高港口辐射能力和国际化水平。全年完成货物吞吐量2亿吨，集装箱运量300万标箱。北海新区以高新技术产业和现代服务业为重点，同步推进园区开发与项目建设，建设创新型经济区。沿海产业基地以电子信息、先进装备制造等高端产业为重点，努力打造冶金及深加工、数字工业创业、新材料新能源、商贸物流四大基地，全年完成固定资产投资150亿元。开发区围绕打造创新产业集聚高地，加大临港和滨海工业园区的项目引进力度，着力构建钢铁及深加工、输变电、食品加工、临港产业四大产业集群，全年完成固定资产投资270亿元，确保主要经济指标增幅保持在30%以上。高新区要加快渤海科技城建设步伐，打造高新技术研发、创意创业、孵化和信息平台，尽快成为产业聚集的“设计师”和成果转化的“加速器”。仙人岛能源化工区要加快能源化工和石化物流产业发展，壮大产业规模，全年完成固定资产投资60亿元。中小企业园要加快推进辽宁青年创业园等专业园区发展，建设充满活力的中小企业创业示范区。

　　打造百里临海景观带。加强沿海生态环境保护，搞好岸线、湿地、海防林保护与开发，加大沿岸山地植被恢复力度，全面推进山体绿化和河流整治，充分彰显滨海自然景色。加快景观带规划建设，规划建设20公里滨海景观大道，实施大清河、沙河景观改造，建设开发区珍珠湾、河海风情园、北海海蚀公园、仙人岛景观大道等区域标志性景观。依托产业集聚和城区发展，实施城区、园区绿化和亮化工程，经过几年的努力，将100公里海岸线打造成为独具特色的沿海风景线。

　　（三）加快发展县域和城区经济，确保“三年倍增计划”扎实推进

　　大力发展现代农业和县域经济。以生态农业、设施农业、品牌农业和观光旅游农业为发展方向，进一步推进农业结构由适应性调整向战略性调整转变、由传统农业向现代都市农业升级。围绕打造“营口大米”、“盖州苹果”、“鲅鱼圈海蜇”等名牌产品，扶持一批竞争优势强、带动作用大的农业产业化龙头项目。全年新增“三品”认定面积10万亩，新创省以上农业品牌15个。加强农业基础设施建设，积极培育农业社会化服务体系，规范推进农村经济合作组织建设，提升农业整体水平。深化农村改革，全面落实强农惠农政策，创新土地流转模式，建立投入多元化的扶持农业农村经济发展新机制。大力发展县域经济，加快工业化、城镇化步伐。全年县域经济生产总值、固定资产投资、财政一般预算收入分别增长27%、35%、30%，确保完成“三年倍增计划”。积极培育新农村典型试点村，以点扩面，带动新农村建设整体水平的不断提高。

　　加快发展服务业和城区经济。坚持把发展服务业作为城区经济的重点，促进传统服务业上水平，实现新兴服务业发展新突破，提升城区经济实力。站前区、西市区要用两年时间完成“三年倍增计划”。推进服务东北的蔬菜、水果及食品物流基地建设，引进国内外知名品牌、填补行业空白的产业项目，促进服务业发展提速、比重提高、层次提升。加快“退二进三”步伐，年内市区搬迁企业10户。加快推进辽宁环渤海钢材城、营口汽贸城等重点项目建设。积极培育服务外包、文化创意等高端服务业，大力发展社区服务和连锁经营，构建现代服务业体系。围绕建设中国北方最佳温泉城市，发挥“山、海、林、泉、寺”的优势，继续开发以温泉度假为龙头的特色旅游产品和冬季旅游项目，实施金牛山古人类遗址公园保护性开发建设，扶持乡村旅游特色村发展，打造旅游产业链，确保全年旅游总收入增长30%以上。进一步规范房地产开发秩序，提高准入条件，提升总体水平，推动房地产业持续健康发展。确保全年服务业总量占经济比重提升两个百分点，城区经济增幅高于全省平均水平。

　　（四）加快产业结构调整，促进工业经济优化升级

　　提升园区专业化水平。围绕工业经济转型升级，完善各园区产业发展规划，明晰主导产业定位。按照专业化的发展方向，确定各园区主打产业，大力推进乐器、汽保等特色园区建设。整合土地资源，优先保障重点项目建设用地，提高土地单位投资强度和开发效益。着力抓好园区基础设施、管理体制和综合服务体系建设，强化项目承载和配套服务能力，努力把园区建成科技创新的示范区、吸纳人才的集聚区、新兴产业发展的核心区。

　　大力发展产业集群。坚持以市场为导向，以技术创新为先导，加快产业结构调整。继续做强镁质材料产业，开发以镁为原料的新产品，打造大石桥市“中国镁都”品牌。抓好冶金、石化等主导产业发展，延伸产业链条，促其向规模化、集群化方向发展。完善中小企业考核和信用评价体系，优化发展环境，促进中小企业做大做强。围绕重点园区、骨干企业、知名品牌和重大项目，积极发展电子信息、新能源、新材料等新兴产业，加速产业优化升级，形成以新兴产业为主导的经济增长点。进一步优化资源配置，推动产业配套和项目拓展，充分发挥行业协会的桥梁纽带作用，为产业集聚创造条件。全市要重点培育1—2个产值1000亿元的产业集群，每个县区、园区都要有1—2个产值达到300—500亿元的产业集群。

　　强化资源节约和综合利用。把节约能源、保护环境、集约用地作为转变发展方式的着力点，发展低碳经济。健全节能减排责任制和考核体系，落实“三同时”和项目准入制度，淘汰落后产能，积极推广节能、资源综合利用技术，确保完成节能减排目标。严格耕地保护制度，进一步规范土地和矿山开发秩序，严禁破坏性开发建设。

　　（五）加强基础设施建设，努力改善城乡环境

　　调整完善规划布局。继续抓好《营口市城市总体规划》修编，做好北海新经济区规划、主城区重点区域控制性详规和城市水、电、路等基础设施规划，全面完成市县两级土地利用总体规划修编。认真搞好重点区域的城市标志性设计，高水平做好高速铁路站区交通枢纽的规划设计。加强规划衔接协调，发挥规划的引导统筹作用，保证规划的科学性和严肃性。

　　加强基础设施建设。启动营口机场前期工程，加快沿海产业基地、仙人岛园区铁路工程进度，搞好城市轨道交通规划论证。推进北海新城、营东新城、沿海产业基地等基础设施配套项目建设。进一步抓好城乡结合部、城市出入口等重点部位的建设改造。积极推广使用建筑领域新能源、新材料，建设环保节能工程。加快推进城市集中供热、生活垃圾处理厂等重点项目建设步伐。以农村供水、水库除险、生态恢复为重点，进一步加强农村基础设施和生态环境建设。全年新增灌溉面积1.2万亩，完成造林绿化面积12.6万亩。认真办好15件重点民生工程，抓好去年确定的“五大惠民工程、十件实事”跨年度续建工作。

　　提高城乡管理水平。理顺和完善城市管理体制，下移管理重心，充分调动城区管理积极性，逐步建立和完善责权利相统一的城市管理体系。以市容市貌和道路交通专项整治为重点，加强城市管理和设施维护，以街巷、居民小区、公共场所为重点，切实解决乱停乱放、乱搭乱建、乱种乱养等群众反映的突出问题。以改善农村人居环境为重点，全面推进镇容村貌集中治理。深入贯彻实施《水污染防治法》，加大对盖州、大石桥境内工矿企业污染源治理，确保城乡饮用水安全。实施大气污染综合整治，加快二氧化硫和化学需氧量减排工程建设，努力创建全国节水型城市和全国卫生城市。

　　（六）加快社会事业发展，不断提高民生质量

　　完善就业和社会保障体系。实施更加积极的就业政策，重点解决城镇低保户、低保边缘户和困难家庭高校毕业生就业，保持“零就业家庭”动态为零，全年实名制就业5万人以上。扩大养老保险覆盖面，以非公有制企业、个体工商户、灵活就业人员、农民工为重点，确保参保人数达到44.2万人。健全覆盖城乡的医疗保障制度，城镇居民参保覆盖面达到90%以上，新型农村合作医疗参合率达到99.3%。完善城乡最低生活保障制度，健全优抚保障机制和社会救助体系，加强以东部山区为重点的扶贫开发，推进社会福利和慈善事业。积极推进住房保障体系建设，全市新建廉租房6.3万平方米。

　　加快科技兴市步伐。加强科技创新、创业、创意载体建设，开展产学研合作，吸引更多的企业、高校、科研院所来营设立研发机构，引导科技人才创办技术密集型企业。进一步完善义务教育投入保障机制，整合职业教育资源，推进营口大学园新校区建设。促进教育均衡发展，办好学前教育和农村义务教育，提升农村师资水平，提高办学质量。

　　发展卫生体育等社会事业。全面推进医药卫生体制改革，基本医疗服务向基层延伸，基本医疗保障向弱势群体倾斜，重大公共服务政府保障。进一步强化重大疾病防控和应对突发公共卫生事件的能力，加大《传染病防治法》的贯彻实施力度，加快建设市中医院、市第一专科医院，推进区域性中心医院建设，改善医疗条件，提高救治水平。加大监管力度，确保食品药品安全。加快公共体育设施建设，开展全民健身活动，促进群众体育、竞技体育和体育产业发展。加强人口和计划生育工作，提高人口素质，稳定低生育水平。

　　推进文明城市建设。以创建全国文明城市为载体，全面加强精神文明建设。按照《全国文明城市测评体系》的要求，认真抓好硬性指标和特色指标的落实。深入开展群众性精神文明创建活动，加强公民道德建设和普法宣传教育，提升市民文明素质。完善公共文化设施，积极培育具有发展活力和市场竞争力的文化产业，推进地区广播电视网络资源整合和数字电视整体转换工作。积极开展双拥共建活动，进一步做好妇女儿童、民族宗教、外事侨务、老龄、气象、地震、档案、史志等各项工作。

　　维护社会和谐稳定。加强社会治安综合治理，依法打击违法犯罪活动，有效开展社会矛盾化解、社会管理创新、公正廉洁执法活动。畅通诉求渠道，认真解决群众的合理诉求。加强安全生产监管和专项整治，防止重特大事故发生。完善应急管理机制，有效预防和处置各种突发事件。

　　（七）加强政府自身建设，提高科学执政能力

　　创新管理，科学理政。进一步转变政府管理方式，将工作重点放在发展经济、改善民生和公共服务上，提高科学发展能力。不断完善重大事项决策机制，推进政府决策科学化、民主化。坚持政务信息公开制度，保障人民群众知情权和监督权。健全政府部门协调配合机制，进一步转变政府职能。

　　完善制度，依法行政。进一步完善接受人大及其常委会法律监督、工作监督和政协民主监督的措施办法，加强与各民主党派、工商联、无党派人士和人民团体的沟通联系，认真办理人大代表建议和政协委员提案。深入贯彻落实《行政许可法》，严格按照法律规定，规范行政行为。加强行政执法监督，改进行政复议，建立健全责任追究和绩效考评制度，使政府各项工作规范化、制度化。

　　转变作风，高效施政。深化行政审批制度改革，减少和规范行政审批程序，完善联审机制。进一步加强行政审批服务中心建设，打造行政服务“绿色通道”。以提高工作效率和服务水平为重点，狠抓机关作风和效能建设。开展行政效能监察，构建完善的效能监督体系，确保政令畅通，运转高效。强化诚信建设，大力倡导埋头苦干的工作作风，提高服务能力和水平。

　　强化监管，廉洁从政。扎实推进惩治和预防腐败体系建设，建立严格的监督制度和权力制约机制，纠正损害群众利益的不正之风。加大政府资金监管力度，确保资金使用安全。坚持厉行节约，最大限度压缩一般性支出，建设节约型政府。加强公务员教育和监督，努力建设一支高素质的公务员队伍。

　　各位代表，立足新起点，抢抓新机遇，实现新跨越，是时代赋予我们的历史责任和光荣使命。让我们紧密团结在以胡锦涛同志为总书记的党中央周围，全面贯彻落实科学发展观，在省委、省政府和市委的领导下，励精图治，开拓创新，为实现全市经济社会又好又快发展，建设更加和谐美好的新营口而努力奋斗！
